# Journeys Through Genomics: Co-Producing Visual Resources to Communicate Patient Experiences

**DOI:** 10.1177/13607804241252528

**Published:** 2024-06-02

**Authors:** Kate Lyle, Susie Weller, Anneke Lucassen

**Affiliations:** University of Oxford, UK; University of Oxford, UK; University of Oxford, UK

**Keywords:** co-production, genomics, illustration, patient experience

## Abstract

*Journeys through Genomics* is a series of illustrations co-produced with patients and families to communicate their experiences of seeking genomic explanations for a health condition and the wider impact on their lives. The resources are embedded within qualitative longitudinal research exploring patient’s experiences of genomic medicine. This research takes place as genomic medicine becomes an integral part of mainstream care within the UK healthcare system. The depiction of genomic medicine often focuses on its technological components and the speed by which genetic code can be analysed, but here, we present a dynamic and situated understanding of the challenges genomic testing presents for patients and families. We describe the process of working with research participants and an artist to co-produce visual resources that illustrate the complexity of participant’s journeys, situating genomic testing within the broader context of their lives. These resources are designed to help future patients, families, and healthcare professionals understand the process, opportunities, and challenges they may face.

*Journeys through Genomics* is a collection of illustrations co-produced with patients and families to communicate their experiences of genomic medicine and illustrate how these experiences are situated within the broader context of their lives. It is embedded within a qualitative longitudinal study (QLR) following patients and their families as they search for genomic explanations for health conditions.

## Background

Genomic medicine is being integrated into routine medical care in the UK, as technological advances improve genome sequencing capabilities at significantly reduced costs. However, while it has enabled some gains in rare disease diagnoses, the complex relationship between genomic data and health and disease is not fully understood, and generating medically relevant findings is often more complex than commonly portrayed. Indeed, our QLR shows a stark contrast between patients’ lived experiences and the uncomplicated representations of genomic medicine often depicted in policy and the media. This discord in narratives highlights the need to communicate the complexities of patient journeys.

With this goal in mind, we developed the illustrations as a communication tool to provide patients, families, and healthcare professionals with a more dynamic and situated understanding of the challenges genomic medicine presents. In partnership with our participants and an artist, we co-produced four illustrations with intended applications in (1) research, (2) healthcare practice, and (3) public engagement. In the following paragraphs, we outline the co-production process and evaluate the value of the illustrations.

## Co-production process

**Figure 1.**
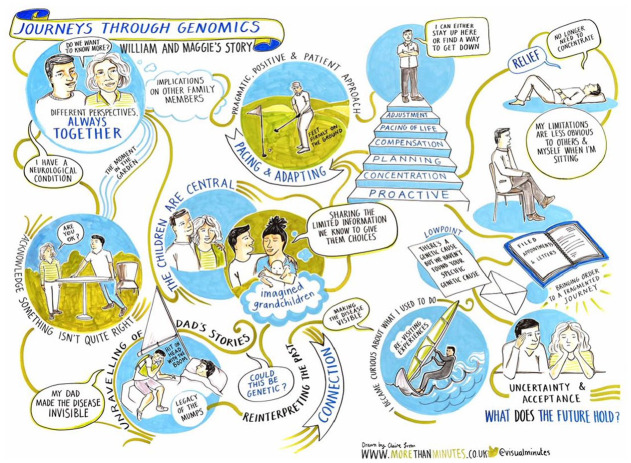
*William and Maggie*. This journey focused on exploring a genetic cause of William’s neurological condition that could have implications for the life courses of their adult children.

**Figure 2.**
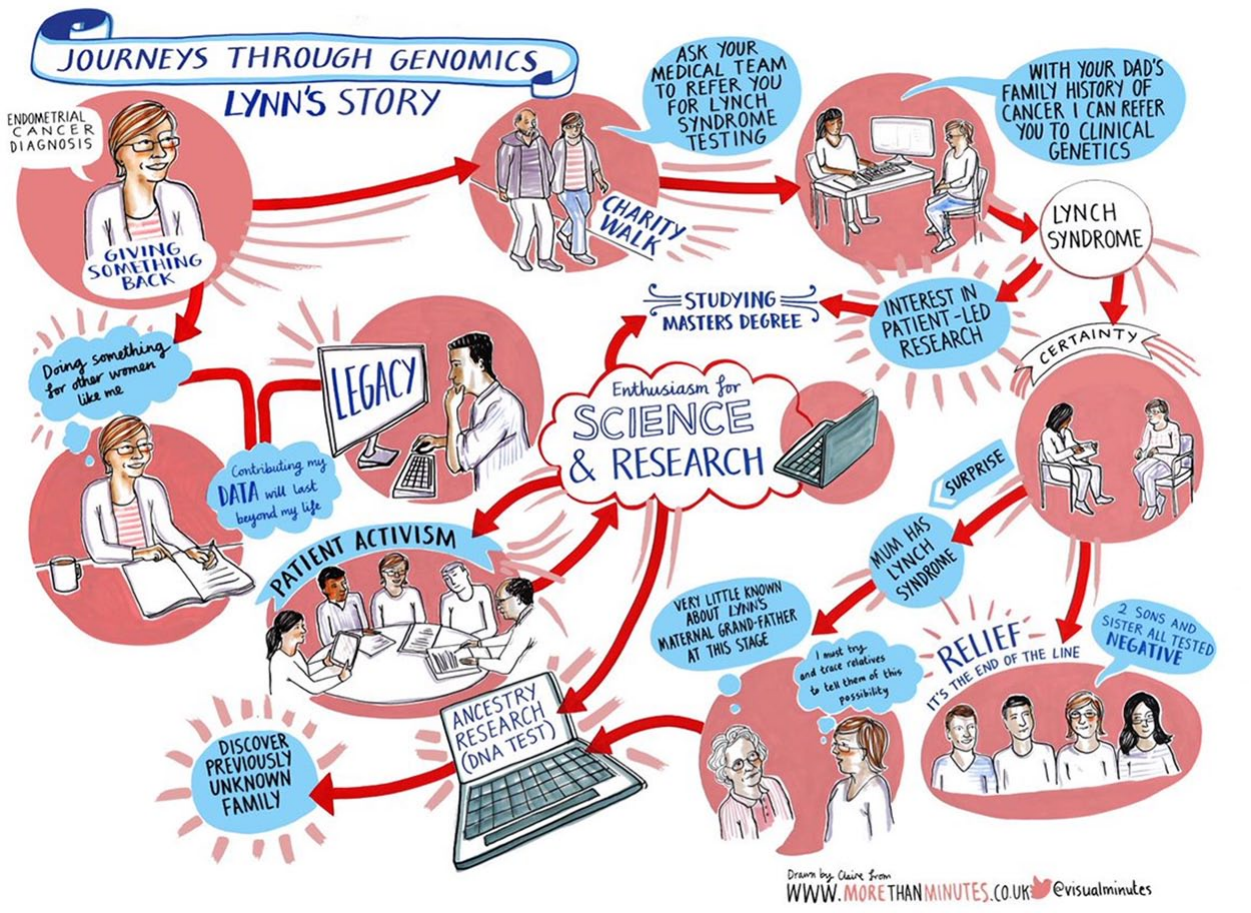
*Lynn*. This journey was shaped by the resources and networks to which Lynn had access, fuelled her passion for research and patient activism, and helped her connect with previously unknown relatives.

**Figure 3.**
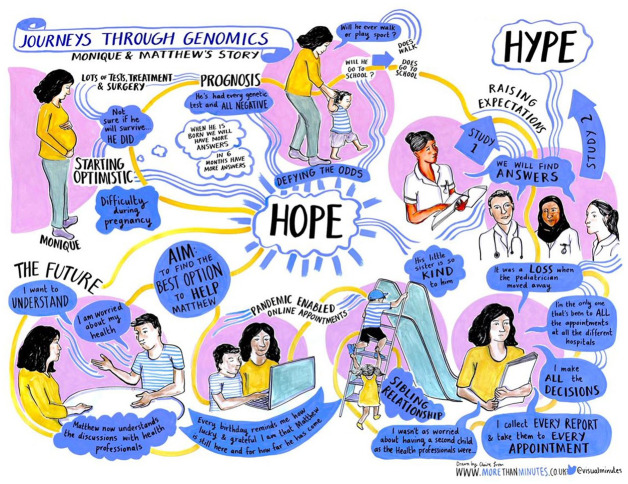
*Monique and Matthew*. This journey focussed on finding an explanation for Matthew’s conditions and explicitly highlights the disconnect between the promises of genomic medicine and her lived experience.

**Figure 4.**
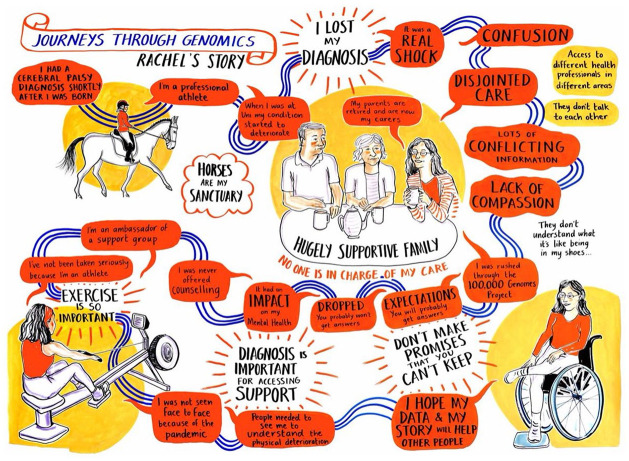
*Rachel*. This journey began after Rachel’s symptoms changed in her 20s and resulted in the ‘loss’ of the diagnosis she had lived with since infancy.

We selected four diverse cases (see Figures 1 to 4) and collaborated with artist Claire Stringer to translate their stories into illustrations. The illustrations were created sequentially, the first serving as a prototype for the overall process. The narratives featured in our QLR interviews provided the focal point for the illustrations, which commenced with KL and SW conducting independent sociological analyses of the interview data.

### Sociological analysis

Using a narrative approach, we employed different sociological lenses to explore the most salient features (e.g. Andrews et al., 2013) of participants’ journeys. For example, Ruth Levitas’ (2013) *Utopia as Method* helped to examine how participants mobilise genomic data to construct different desirable futures. These visions are born out of collective notions of self – what Gilligan (1982) calls a self constructed-in-relation to others – as the heritability of genomic data draws participants to position themselves in relation to past, present, and future generations. The story of William and Maggie (Figure 1) provides an apt example. Many years ago, William’s father displayed similar symptoms to those now affecting William. In the past, different family stories were used to explain his father’s condition, but with the emergence of William’s symptoms, these narratives unravelled, replaced by a genetic understanding. Now William and Maggie continue the search for a genetic explanation, in the hope of enabling their children and future grandchildren a level of agency over their future that William did not experience himself. This narrative is depicted in the illustration, with William’s father featuring in the bottom left and his children and future grandchildren at the centre, symbolising the generational impact, and William and Maggie’s sense of self-in-relation to other generations.

Employing the concept of linked lives from the life course theory (Elder, 1994) facilitated a relational approach in considering who constitutes the patient. Although an individual may undergo genetic testing to explore an aspect of their own health, any outcomes may also have implications for their relatives. Through the co-production process, the salience of more collective, inter-generational views of patienthood emerged. For instance, having been diagnosed with cancer, Lynn’s journey through genomics (Figure 2) not only involved a quest for a potential genetic explanation for her ill health but also a reconsideration of the ensembles of relationships that might be affected. With a diagnosis of Lynch syndrome (which increases the risk of some cancers), Lynn was originally concerned about the future health of her sibling and children, as depicted in the bottom right of the illustration. She also looked to the past to consider the implications for other family members, which led to some difficult family discussions about her estranged paternal grandfather. Lynn has since connected with previously unknown relatives, passing on information about potential heritable risks, as depicted by the laptop. In this way, our research highlights the discord between participants’ collective understandings of patienthood Weller et al. (2022) and more individualised notions common in medical discourses.

### Creative process

In an online meeting, we shared our interpretations of patient stories with Claire, who observed our discussion of a specific case and later translated them into a visual representation. Claire started the creative process by focusing on a visual ‘hook’ that resonated with the narrative, such as the playground in the story of Monique and Matthew (Figure 3). Claire shared an initial sketch with us and incorporated our feedback, before presenting a second iteration for participant input.

KL and SW met with each participant online, first sharing the sketch and discussing their immediate responses and ideas. Participants then had time to reflect on a digital copy before we arranged a second meeting to discuss the content, imagery and layout. Participants considered the illustrations in different ways. Lynn, for instance, focused on chronological accuracy, cutting out and rearranging different components of the sketch. Others, such as Monique and Rachel, concentrated on refining language or visual elements. Claire altered the sketch based on participants’ contributions and, after multiple rounds of revisions, converted the sketch into a final illustration once all parties were satisfied. Thus, the evolving process combined the QLR work with participants, illustration techniques, and working collaboratively with participants.

## Evaluation

In the following sections, we reflect on the value of the illustrations against the three applications we envisaged for them.

### Research tool

The illustrations are a valuable tool for our QLR, with the co-production process enabling a deeper understanding of participants’ experiences. In our discussions with participants to re-work the illustration, we delved deeper into their stories as they provided further updates and new reflections. The experience enriched their accounts and enhanced our comprehension of previously discussed issues.

### Healthcare tool

The illustrations have practical healthcare applications. We presented the first illustration to the Participant Panel, an advisory group of participants in Genomics England. Their feedback was overwhelmingly positive, with enthusiasm for using the illustrations to prepare future patients and healthcare professionals for the challenges they may face on their journey.

### Public engagement

We integrated the illustrations with a hands-on activity at a science festival to demonstrate the complexities of interpreting genomic data and the challenges faced by patients. They brought to life the complexity of genomics, as specific elements, such as the importance of family tales in William and Maggie’s story or the impact of losing a diagnosis in Rachel’s account, which resonated with the audience. In this way, the illustrations added a compelling dimension to the activity, making the complexities of genomic medicine more accessible and relatable to a broader audience.

## Ethical considerations

Guided by an ethic of care (c.f. Lyle et al., 2022), we prioritised protecting participants’ anonymity due to the familial implications of genetic data, particularly those living with rare diseases. We were also keen to avoid overburdening participants, many of whom were already balancing work, family life, and their health. Co-producing illustrations provided a way to ensure participant’s complex stories were not oversimplified or caricatured, giving them control over their level of involvement. We were moved by participants’ overwhelming enthusiasm to share their experiences in this way, driven by a genuine desire to assist future patients and families.

The process of co-producing the illustrations was emotionally intense for both our participants and us as researchers, a dimension we initially overlooked. Presenting the initial sketch to participants for the first time triggered a strong emotional response that we were unprepared for. We had been so focused on *how* to represent a patient’s journey that we overlooked the emotional weight of *what* was represented – every difficult decision or painful experience over multiple years condensed on a sheet of paper. This initial experience taught us not to expect participants to absorb everything in one meeting. We realised the importance of carving out time and space for reflection, along with multiple iterations of changes, to ensure a more considerate co-production process.

## Conclusions

Our patient journeys illustrations contribute to the array of time-aware visual methods employed to generate data, often as part of a participatory approach. Some use illustration, images, or objects to elicit stories, while others are diagrammatic. For example, Tinkler et al. (2021) describe biographical mapping, which involves using photos and artefacts to evoke memories and foster reflection, resulting in collages designed to represent encounters, places or journeys. Worth’s (2011) focus is on life-mapping and the co-construction of diagrammatic representations of young people’s transitions to adulthood. Other forms of graphic communication include relational mapping where the emphasis is on representing visually emotional proximity/distance to others (Hanna and Lau Clayton, 2012; Weller et al., 2011). These approaches, and ours, are united by the dual commitment to emphasising the non-linearity of journeys and the involvement of participants in the process. To enhance their accessibility, we are working with participants to develop animated illustrations accompanied by narration.
